# Prevalence, evolution, and related risk factors of kidney disease among Spanish HIV-infected individuals

**DOI:** 10.1097/MD.0000000000007421

**Published:** 2017-09-15

**Authors:** Javier Juega-Mariño, Anna Bonjoch, Nuria Pérez-Alvarez, Eugenia Negredo, Beatriu Bayes, Josep Bonet, Buenaventura Clotet, Ramon Romero

**Affiliations:** a Servicio de Nefrología, Hospital Germans Trias i Pujol, Badalona; b Universitat Autónoma de Barcelona; c Unitat VIH, Fundació Lluita contra la SIDA, Servicio de Medicina Interna, Hospital Germans Trias i Pujol, Badalona; d Universitat de Vic-Universitat Central de Catalunya, Barcelona; e IrsiCaixa Foundation, Badalona, Spain.

**Keywords:** chronic kidney disease, epidemiology, HIV, human immunodeficiency virus, tenofovir

## Abstract

Prevalence of kidney disease (KD) is increasing among human immunodeficiency virus (HIV)-infected population. Different factors have been related, varying on different published series.

The objectives were to study prevalence of KD in those patients, its evolution, and associated risk factors.

An observational cohort study of 1596 HIV-positive patients with cross-sectional data collection in 2008 and 2010 was conducted. We obtained clinical and laboratory markers, and registered previous or current treatment with tenofovir (TDF) and indinavir (IDV). The sample was divided according to estimated glomerular filtration rate (eGFR) by modification of diet in renal disease (MDRD) equation. Group 1: eGFR ≤60 mL/min/1.73 m^2^; group 2: eGFR >60 mL/min/1.73 m^2^.

Among the patients, 76.4% were men, mean age (SD) 45 ± 9 years, time since diagnose of HIV 14 ± 7 years, and 47.2% of the patients received previous treatment with TDF and 39.1% with IDV. In 2008, eGFR ≤60: 4.9% (91.4% of them in chronic kidney disease [CKD] stage 3, eGFR 59–30 mL/min); this group was older, presented higher fibrinogen levels, and more patients were treated previously with TDF and IDV. In 2010, eGFR ≤60: 3.9% (87.1% stage 3 CKD). The 2.4% of cohort showed renal improvement and 1.3% decline of renal function over time. The absence of hypertension and treatment with TDF were associated with improvement in eGFR. Increased age, elevated fibrinogen, decreased albumin, diabetes mellitus, hyperTG, and worse virological control were risk factors for renal impairment.

The HIV-positive patients in our area have a CKD prevalence of 4% to 5% (90% stage 3 CKD) associated with ageing, inflammation, worse immune control of HIV, TDF treatment, and metabolic syndrome.

## 1 Introduction

The course and prognosis of human immunodeficiency virus (HIV)-positive patients has radically changed since the development of the highly active antiretroviral therapy (HAART), leading to increased patient survival and lower morbidity.^[1]^ Recent data showed an increasing prevalence of kidney disease (KD) in these patients compared with the general population, being related to increased mortality and morbidity.^[2–4]^ The involved factors were as follows: a direct effect of the virus itself, closely related to the immune status; prolonged use of antiretroviral therapy (ART) (tenofovir [TDF], indinavir [IDV], and others); frequent use of concomitant therapy with nephrotoxic drugs; increase of comorbidities such as diabetes mellitus (DM), dyslipidemia (DLP), and hypertension; high prevalence of coinfection by hepatitis B and C compared with general population. All these factors now turn KD an entity of importance in our population that impacts on their short and long-term prognosis.^[5–11]^

Previous studies have examined the prevalence of KD among the population infected with HIV-1, showing large variations due to differences in racial distribution, clinical characteristics, and immunological control.^[12,13,11]^

### 1.1 Objectives

To determine the prevalence of KD in the population infected by HIV-1 in our environment and stratify it according to the criteria of the kidney disease outcomes quality initiative (K/DOQI) 2002 classification,^[14]^ and to establish its evolution over time and potential risk factors for KD development in such patients.

## 2 Materials and methods

Cross-sectional and retrospective unicentric study of 1596 patients with HIV-1 controlled in our center was conducted. Information about HIV-infected patients followed by the HIV unit of our center, Germans Trias i Pujol hospital at Badalona (Barcelona), Spain, was obtained. More than 3500 HIV-infected patients are currently controlled by our HIV unit, with large experience in research, clinical studies, and international publications.

Review and consent from a local ethics committee was not required as the study was designed as a noninterventional, descriptive, and retrospective study, containing information obtained from global blood test results database and available regular clinical notes. The study was reviewed and approved by the head of HIV unit and head of Nephrology Service at Germans Trias i Pujol Hospital.

Two cross-sectional data were collected from those patients among the general HIV cohort controlled in our center who were consecutively visited and had a blood test performed in the outpatient clinic. Baseline data were gathered from those visited between October and December of 2008, and final data were obtained from the same patients visited again between August and October of 2010 to explore the evolution of this cohort and compare baseline versus final data.

There were 16 patients who were lost to follow-up among the group that showed eGFR >60 mL/min at baseline. We detected 7 deaths among those with KD (eGFR ≤60 mL/min) at baseline and 1 death among those who presented decline in renal function over time during the observation period. In those 8 cases, last available data within the observation period were collected as final cut.

In each section multiple data were obtained regarding the following:

1. Clinical characteristics: age, sex, duration of HIV infection, coinfection hepatitis B virus (HBV), hepatitis C virus (HCV), and syphilis serology; cardiovascular risk variables and metabolic disorder were defined according to the criteria of metabolic syndrome NCEP ATP-III 2001^[15]^ and review by the American Heart Association in 2005.^[16,17]^ Impaired fasting glucose (IFG) was defined as blood glucose ≥5.6 mmol/L and DM if blood glucose was ≥7 mmol/L.^[18]^
2. Analytical data: hemoglobin, fibrinogen, albumin, total cholesterol, high-density lipoprotein (HDL), low-density lipoprotein (LDL), triglycerides (TGs), calcium and phosphate levels, HIV viral load (VL), total lymphocytes, and CD4 and CD8 (absolute and percentage). Kidney function was measured by values of plasma creatinine, urea, eGFR MDRD-4 IDMS (eGFR = 175 × [Cr/88.4] × 1154 [age] − 0203 [×0.742 if female]; [×1.210 if black]).^[14,19–21]^ It was encoded as a categorical covariable CD4 count ≤ or >200 cells/mL, undetectable VL was considered as VL ≤50 copies/mL, and cut-off VL > or ≤400 copies/mL and VL > or ≤4000 copies/mL were stablished. The latter cut-offs have shown relationship with impaired renal function in previous studies.^[5,6,9,11]^
3. Treatment with TDF and/or IDV. These variables were encoded by comparing those who had received indistinctly prior or contemporaneous treatment with those who had never received those drugs.

Kidney disease was defined as one eGFR estimated by MDRD-4 IDMS ≤60 mL/min/1.73 m^2^ in the baseline survey. The sample was divided into 2 groups: group 1 (KD): GFR ≤60 mL/min/1.73 m^2^; and group 2 (normal renal function): GFR >60 mL/min/1.73 m^2^.

Group 1 was stratified by different stages of KD. Groups 1 and 2 were compared based on the variables obtained on each of the cross-sectional analysis made.

Additional data about group 1 in the baseline (2008) regarding clinical diagnosis of high blood pressure, hypertension (HBP), DLP, and DM, and also body mass index (BMI), smoking habit, dialysis requirement, and mortality rate were obtained.

This group was subdivided according to the evolution of renal function during the observation period. Group A—patients who persisted showing KD in baseline and final analysis; group B—those who showed improvement of renal function (GFR ≤60 mL/min/1.73 m^2^ at baseline and eGFR >60 mL/min/1.73 m^2^ at final cut), comparing them.

**Figure 1:**
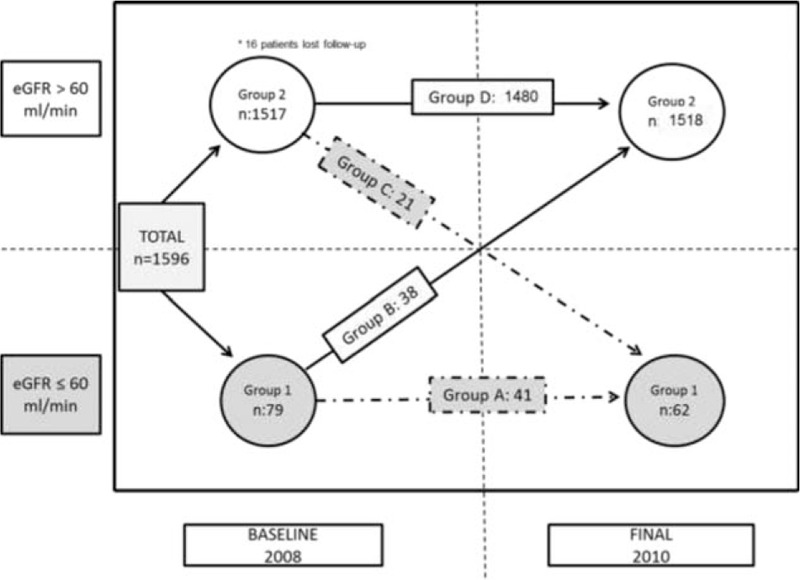
Distribution groups and temporal evolution of the population.

On the contrary, group 2 (preserved baseline renal function) was subdivided into group C normal baseline eGFR with progressive impairment of renal function; and group D eGFR always preserved, comparing both groups (Fig. 1).

Group C clinical data were obtained regarding the diagnosis of hypertension, DLP, DM, and smoking, and withdrawal of previous treatment with TDF and/or IDV during observation period was encoded as a new variable. Requirement of renal replacement therapy techniques and death during the observation period were recorded.

### 2.1 Statistical analysis

The association analysis between qualitative variables was performed using the Pearson chi-square test. Student *t* test, Mann–Whitney *U* test and Wilcoxon test were used to compare continuous variables. A multivariate logistic regression model including statistically significant variables was developed. A significance level of 5% (*P* ≤ .05) in hypothesis testing was adopted. SPSS (version 15.0) statistical package was used. (SPSS, Inc., Chicago, IL).

## 3 Results

In all, 1596 Caucasian patients were included in the study, among which 76.4% were men, with a mean age of 45 ± 9 years, the average time of infection was 14 ± 7 years, and undetectable VL 76.5% in 2008. Also, 47% had received previous or current treatment with TDF (Table 1).

**Table 1:**
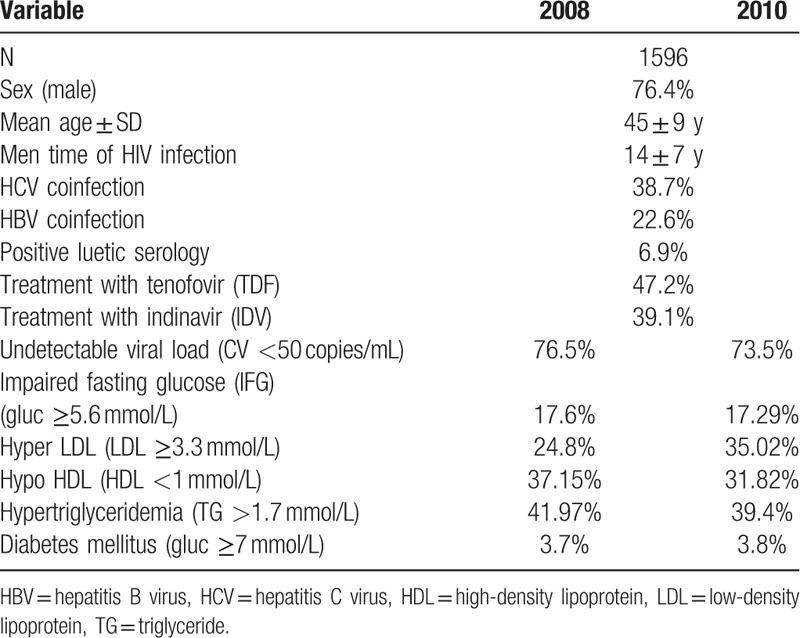
General characteristics of the cohort.

In 2008, group 1 (KD) was 4.9% of the total, having an average eGFR of 49.09 ± 12.08 mL/min/1.73 m^2^. The KD stageswere as follows: stage 3 (eGFR ≤60–30 mL/min) 91.1%; stage 4 (GFR <30–15 mL/min) 5.1%; stage 5 (GFR >15 mL/min) 3.8%.

The 79 patients in group 1 (KD) had important prevalence of DLP (62.8%), hypertension (26.9%), DM (15.4%), and smoking (44.9%). They received in large proportion previous or current treatment with TDF (73.4%) and IDV (59.5%). The 3.8% of these patients required starting hemodialysis. A mortality rate of 8.9% was observed during the observation period.

When comparing in baseline cut (2008) group 1 versus group 2, patients with KD showed statistically significant higher age, fibrinogen, and percentage of VL >400 copies/mL, and had received previous or current treatment with TDF and IDV in greater proportion. No significant differences between lipid levels, immune status, time of HIV infection, or prevalence of coinfection with HCV and HBV were observed. Higher prevalence of IFG and DM was observed in patients with worse renal function, without reaching statistical significance.

In 2010, the prevalence of KD was 3.9% of the total, with average 45.4 ± 14.2 eGFR mL/min/1.73 m^2^. KD distribution stages were as follows: stage 3—87.1%; stage 4—6.5%; stage 5—6.5%.

In 2010, group 1 showed statistically significant higher age, higher TG levels, and fibrinogen; and lower levels of albumin and hemoglobin; higher percentage of CD4 <200 cells/μL; lower CD4 absolute and percentage, and total lymphocyte when compared with group 2. There were no significant differences between the percentage of patients who had received prior or current treatment with TDF or with IDV, or in prevalence of coinfection with HCV or HBV.

In addition, in the final cut, significant differences between groups in prevalence of DM, IFG, and hypertriglyceridemia (HTG) were obtained. In all cases, higher proportion of metabolic disorders was observed in the group of impaired renal function (Table 2).

**Table 2:**
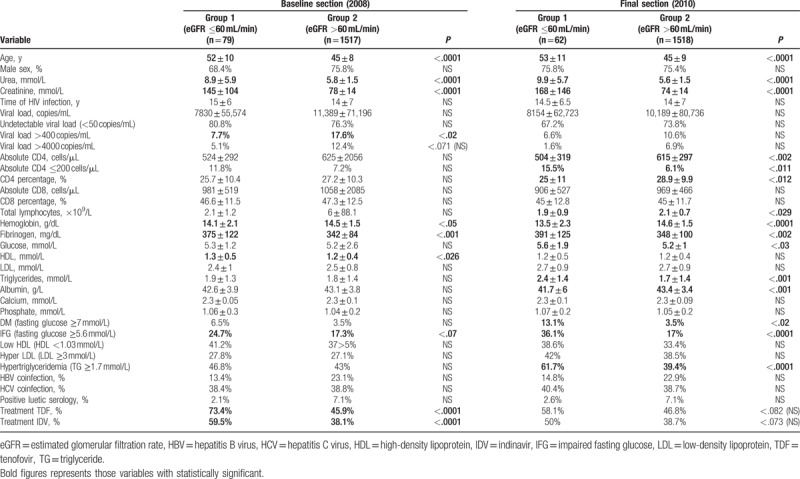
Differences group 1 versus group 2 in basal (2008) and final (2010).

### 3.1 Evolution of cohort's renal function

Regarding the evolution of renal function during the study period, we observed the following: patients showing persistent KD, remaining in group 1 (maintained eGFR ≤60 mL/min/1.73 m^2^) 2.6%; those with stable preserved renal function, remaining in group 2 (maintained eGFR >60 mL/min/1.73 m^2^) 92.7%, improvement of renal function observed in 2.4% of patients, and 1.3% showed decline of renal function during observation period.

Up to 47.2% of group 1 patients showed improvement in renal function, reaching GFR >60 mL/min, whereas 52.8% continued showing eGFR ≤60 mL/min (Fig. 1).

The evolution of renal function within group 1 was studied by comparing those who persisted showing renal disease (group A), and those who showed improvement of renal function to levels of GFR >60 mL/min/1.72 m^2^ (group B).

Regarding clinical variables, patients with improvement in renal function showed lower prevalence of hypertension (13 vs 40%) and higher percentage of previous or current treatment with TDF (86.6% vs 61%) statistically significant. Higher prevalence of factors of metabolic syndrome and cardiovascular risk (diagnosis of DM, DLP, and HTG) in those patients without improvement in renal function and without reaching statistical significance was observed. There were no significant differences between age, smoking habit, time of HIV infection, or coinfection with HBV or HCV.

As for the analytical variables, in 2008, the group B (improvement of renal function) showed lower levels of TG and lower prevalence of CD4 <200 cells/μL, which is statistically significant.

In 2010, group B had higher levels of hemoglobin, which is statistically significant, without other relevant differences between the 2 groups (Table 3).

**Table 3:**
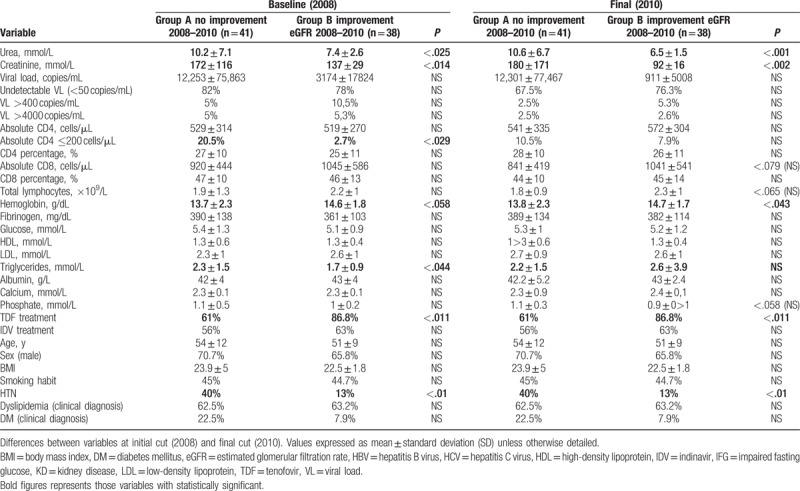
Comparison baseline group 1 (subgroup B [renal function improvement] vs subgroup A [remaining KD]).

Subsequently, the group of patients with normal renal function in the baseline (group 2) was divided according to progression of renal function in group C: declining renal function, from normal GFR in the baseline to KD in the final cut; and group D: patients with maintained normal renal function over time.

We reviewed clinical records of patients in group C (n = 21) obtaining additional data. These patients were mostly male (85.7%), mean age 53 ± 11 years, and had a higher prevalence of cardiovascular risk factors (hypertension 40%, DM 70%, DLP 25%) and 55% of HCV coinfection. Among them, 52.4% had received prior or current treatment with TDF, which was withdrawn after deterioration of renal function in 30% of them. Hemodialysis requirement was observed in 10% (2 patients), with a mortality of 5% (1 patient) during the observation period.

In 2008, patients in group C showed statistically significantly higher age and worse infective/immune values with a higher prevalence of VL >4000 copies/mL, and lower levels of CD4 percentage, CD4 absolute, and higher prevalence of CD4 ≤200 cells/mL.

Group C had also higher levels of fibrinogen and lower levels of albumin and hemoglobin, and a higher proportion of IFG, DM, and HTG, all statistically significant.

In 2010, the significant differences obtained largely coincided with those observed in the baseline cut mentioned above (Table 4).

**Table 4:**
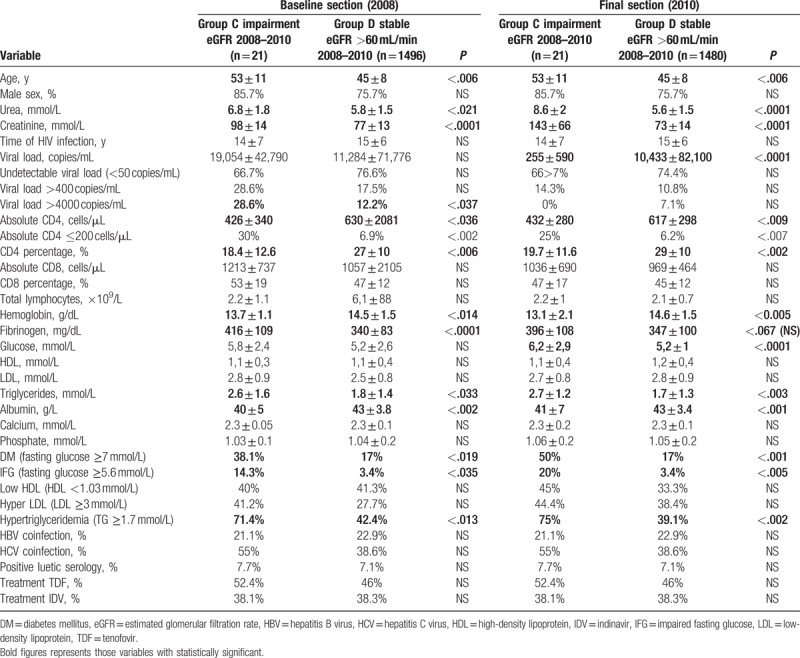
Comparison basal group 2 (subgroup C [initial normal eGFR, decline to FG ≤60 mL/min] vs subgroup D [stable eGFR > 60 mL/min]).

### 3.2 Multivariate analysis

Multivariate analysis was performed, obtaining as independent markers of KD in 2008: older age (odds ratio [OR] 1.079, 95% confidence interval [CI] 1.050; 1.109, *P* < .001), elevated levels of fibrinogen (OR 1.004, 95% CI 1.002; 1.006, *P* < .004), and previous or current treatment with TDF (OR 2.732, 95% CI 1.575; 4.739, *P* < .001).

Independent markers of KD in final cut 2010 were as follows: older age (OR 1.099, 95% CI 1.067; 1.131, *P* < .001), lower albumin (OR 0.931, 95% CI 0.869; 0.997, *P* < .04), and HTG (OR 2.721, 95% CI 1.497; 4.947 *P* < .001). DM approaches statistical significance (*P* < .053).

Older age (OR 1.093, 95% CI 1.061; 1.126, *P* < .001) and elevated levels of fibrinogen (OR 1.004, 95% CI 1.002; 1.006, *P* < .001) were the only independent risk factors for the development of KD present in both baseline and final cuts.

The absence of hypertension (OR 0.212, 95% CI 0.061; 0.733, *P* < .014), lower TG levels (OR 0.592, 95% CI 0.366; 0.956, *P* < .032), and current or previous treatment with TDF (OR 6.271, 95% CI 1.710; 23, *P* < .006) were independent markers of renal function improvement over observation period both in baseline and final sections.

Independent markers of declining renal function over time according to baseline 2008 data were older age (OR 1.102, 95% CI 1.045; 1.162, *P* < .001), elevated fibrinogen (OR 1.005, 95% CI 1.001; 1.009, *P* < .014), and presence of VL >4000 copies/mL (OR 3.397, 95% CI 1.058; 10.905, *P* < .04) were established as markers of declining renal function during observation period. CD4 percentage levels had a protective effect (OR 0.941, 95% CI 0.892; 0.992, *P* < .025).

According to final data in 2010, independent markers of declining renal function over time were older age (OR 1.084, 95% CI 1.024; 1.148, *P* < .005), HTG (OR 5.894, 95% CI 1.844; 18.846, *P* < .003), and DM (OR 3.618, 95% CI 1.301; 10.066, *P* < .014). Higher levels of CD4 percentage had again protective effect (OR 0.920, 95% CI 0.873; 0.970, *P* < .002).

In summary, both in 2008 and in 2010, we found higher age, elevated markers of inflammation, worse infective and immune status, and presence of cardiovascular risk factors to be independent markers of impaired renal function in HIV-positive patients in our environment.

## 4 Discussion

In our study of a large cohort of HIV-infected patients, the prevalence of KD was between 3.9% and 4.9% over the period 2008 to 2010. There was a predominance of moderate stage of KD, as 91% to 87% of patients showed stage 3 chronic kidney disease (CKD).

The spectrum of renal disease in HIV-positive patients seems to have changed in recent years, with increased relevance of toxic effect of antiretrovirals (ARVs), the inflammatory status of these patients, or the increasing emergence of cardiovascular risk factors common to the general population as important factors leading to kidney function impairment.^[8]^

Our results are consistent with some of the recent work in Europe regarding the prevalence of KD in patients with HIV infection.^[13,11]^ Other studies show highly variable prevalence according to the social or sanitary level, clinical features, treatment, and racial distribution.^[12]^

The EPIRCE (Epidemiología de la Insuficiencia Renal Crónica en España) 2010 study conducted on general population in Spain showed a percentage of stage 3 to 5 CKD of 6.8%, although in the age segment similar to our sample, aged 40 to 64 years, the prevalence of KD 3 to 5 was of 3.3%.^[22]^ These data suggest that among HIV-infected patients of our environment, the prevalence of CKD grade 3 to 5 is slightly higher than that in the general population.

As for the associated factors, advanced age is a well-known risk factor for development of KD, and also other traditional factors of cardiovascular risk (DM, HTN, and DLP).^[14–18]^

We must also consider what some authors named the “premature aging” among this population, from a cardiovascular perspective, conditioned by various factors related to the increase in life expectancy since the beginning of the HAART era.^[23]^

The ARV treatment plays an important role in those complex processes. Interrupting HAART studies have shown increased cardiovascular mortality after discontinuation of treatment, but other studies related some ART drugs such as IDV with increased cardiovascular risk.^[24,25]^

Several publications have studied various inflammatory markers in HIV-positive individuals like fibrinogen, C-reactive protein (CRP), interleukin (IL)-6, D-dimer, and others.^[26–34]^ HIV infection can activate different inflammatory pathways of the vascular wall with cytokine release and endothelial adhesion molecule expression. It has been established that HIV-positive patients have higher levels of fibrinogen than healthy controls. High levels of other inflammatory markers such as CRP have been related with mortality in this population, even with CD4 counts >500 cells/μL. It has been suggested that such pro-inflammatory environment might favor the development of cardiovascular disease.^[26,35–38]^

In our study, we observed raised fibrinogen levels and lowered albumin levels as markers of inflammation independently associated with the prevalence of KD. Fibrinogen was also related to decline of renal function, what would correlate higher inflammatory status of these patients with renal function impairment.

Most population studies include hypertension and/or DM as independent risk factors for the development of KD in HIV-infected patients, similar to the general population.^[5,6,9–11,39,40,41]^ We observed an increasing prevalence of cardiovascular risk factors common to the general population in our cohort of HIV-positive individuals, with subsequent renal involvement. We found that hypertension is an independent marker of no improvement in renal function in patients with GFR <60 mL/min at baseline, and also the presence of DM and HTG were independent markers of renal function impairment among patients with normal eGFR at baseline. This highlights the emergence of HTG as a marker of renal function deterioration in the final cut, but not at baseline, suggesting a higher prevalence of metabolic syndrome and a possible cumulative role of this metabolic disorder over time.

We also explored the current or previous treatment with TDF, as many studies established potential nephrotoxic effect of TDF, IDV, and other ARVs.^[5–42,43,44]^ TDF can cause renal impairment in various forms: tubular disorder, acute, or chronic renal failure, and maintained renal dysfunction after drug withdrawal in some cases.^[7,42,12]^

In the subgroup of patients who showed decline in renal function despite normal eGFR at baseline, more than half had received TDF. This drug was withdrawn in 30% of them. However, the evolution of renal function was unfavorable.

In 2008, treatment with TDF was independent marker of impaired renal function, but in patients who at baseline had eGFR ≤60 mL/min receiving prior or current treatment with TDF was an independent marker of improvement in renal function in the final cut. Based on the pattern observed in group C, and clinical practice, a possible explanation for the double jarring significance of TDF in our study would be the change in medication.

Probably by detecting renal impairment and therefore conducting a closer monitoring, physicians in charge have adjusted or withdrawn the drug to a portion of these patients. For those who responded favorably and recovered renal function, it would thus become a false marker of “improvement” of renal function. The cumulative treatment with TDF could also influence the results.

Other independent markers of renal function impairment obtained were CD4 percentage decline and the presence of VL >4000 copies/μL, regarding the immune status of patients. Some markers of different immune status, such as CD4 absolute, undetectable HIV viral load or CD4 <200 cells/mL, did not obtain statistical significance in our multivariate analysis. Multiple previous studies related deterioration of renal function in HIV-positive patients with impaired immune parameters of disease control.^[5,6,9–11]^

Possible other risk factors for deterioration of renal function as coinfection with HCV, HBV, HIV evolution time, or sex, present in other studies^[5,6,9–11]^ showed no statistically significant difference in our cohort.

### 4.1 Limitations of the study

The study shows the limitations of its design as the transverse observational studies may lack data on some variables.

Therefore, we only have an analytical determination on each of the cross-sections, which might affect the definition of DM and IGT, established from basal glucose levels, requiring confirmation. Detailed information on ART cumulative dose or changes on medication was not available, except in a small group of patients. This is a particularly important aspect, and would confirm our hypothesis about the significance of the treatment with TDF and its withdrawal on this population.

We did not obtain data on proteinuria or impaired urine of patients due to lack of sample.

At the time of obtaining the analytical determinations, the CKD-EPI (chronic kidney disease epidemiology collaboration) formula was not available, as it was developed and implemented in our area from the year 2011, so the stratification of renal function has been made based on the MDRD-4 formula.

No data were available on clinical diagnosis of cardiovascular risk factors about the entire sample, such as the prevalence of hypertension, diabetes, or DLP, although we were able to register these variables in those patients showing KD.

Therefore, further studies are needed with proper prospective, long-term design, to continue development of the study of KD in HIV-positive patients.

## 5 Conclusions

Among the HIV-positive patient population of our country, the prevalence of renal disease, defined as eGFR ≤60 mL/min/1.73 m^2^, was between 3.9% (baseline) and 4.9% (final); 89% of them were in stage III of K/DOQI: eGFR 60 to 30 mL/min/1.73 m^2^. This represents a KD prevalence higher than in the Spanish general population of the same age.

Independent markers of renal impairment were higher age and elevated fibrinogen in both baseline and final cuts, elevated fibrinogen and previous or current treatment with TDF in the baseline 2008, and HTG and decreased albumin in the final cut of 2010.

Independent markers of improvement in renal function in patients with altered initial eGFR: previous or current treatment with TDF and absence of hypertension. On the contrary, independent markers of renal function impairment among patients with preserved initial eGFR were as follows: age, elevated fibrinogen, presence of HTG and DM, CD4 (%), and presence of VL >4000 copies/μL.

No significant associations between KD and prevalence of viral coinfections, time of HIV infection, and sex of patients were found.

These results highlight the potential multifactorial etiology of KD among HIV-infected population. A global perspective and therefore preventive and treatment strategy should focus on all the involved factors.

A majority of the observed renal impairment would present in a subclinical manner, so it is of vital importance that a regular screening and intervention on cardiovascular risk factors, early detection, and adjustment of treatment be done.

Urinary albumin and protein excretion should be regularly performed, and also other urinary tests, looking for tubular injury such as phosphate excretion, calcium excretion, and glucose in urine, all of them possible markers of ART toxicity.

Close cooperative work among nephrology and infectious disease specialists is required, and early referral to renal clinic is recommended to further explore the etiology and possible treatment of the underlying process.

In summary, from our results, the deterioration of renal function in HIV-positive population of our country is related with age, inflammatory state, poor immunological control, antiretroviral therapy (TDF), and markers of cardiovascular risk associated with metabolic syndrome (HBP, DM). All are objectified factors that can be monitored, and potentially most of them are treatable, which is key for detection and early treatment of impaired renal function in this population, resulting in a decrease in cardiovascular morbidity and mortality in these patients.
